# Animal models of respiratory syncytial virus infection

**DOI:** 10.1016/j.vaccine.2016.11.054

**Published:** 2017-01-11

**Authors:** Geraldine Taylor

**Affiliations:** The Pirbright Institute, Ash Road, Pirbright, Woking Surrey GU24 0NF, United Kingdom

**Keywords:** Respiratory syncytial virus, Animal models

## Abstract

Human respiratory syncytial virus (hRSV) is a major cause of respiratory disease and hospitalisation of infants, worldwide, and is also responsible for significant morbidity in adults and excess deaths in the elderly. There is no licensed hRSV vaccine or effective therapeutic agent. However, there are a growing number of hRSV vaccine candidates that have been developed targeting different populations at risk of hRSV infection. Animal models of hRSV play an important role in the preclinical testing of hRSV vaccine candidates and although many have shown efficacy in preclinical studies, few have progressed to clinical trials or they have had only limited success. This is, at least in part, due to the lack of animal models that fully recapitulate the pathogenesis of hRSV infection in humans. This review summarises the strengths and limitations of animal models of hRSV, which include those in which hRSV is used to infect non-human mammalian hosts, and those in which non-human pneumoviruses, such as bovine (b)RSV and pneumonia virus of mice (PVM) are studied in their natural host.

Apart from chimpanzees, other non-human primates (NHP) are only semi-permissive for hRSV replication and experimental infection with large doses of virus result in little or no clinical signs of disease, and generally only mild pulmonary pathology. Other animal models such as cotton rats, mice, ferrets, guinea pigs, hamsters, chinchillas, and neonatal lambs are also only semi-permissive for hRSV. Nevertheless, mice and cotton rats have been of value in the development of monoclonal antibody prophylaxis for infants at high risk of severe hRSV infection and have provided insights into mechanisms of immunity to and pathogenesis of hRSV. However, the extent to which they predict hRSV vaccine efficacy and safety is unclear and several hRSV vaccine candidates that are completely protective in rodent models are poorly effective in chimpanzees and other NHP, such as African Green monkeys. Furthermore, interpretation of findings from many rodent and NHP models of vaccine-enhanced hRSV disease has been confounded by sensitisation to non-viral antigens present in the vaccine and challenge virus.

Studies of non-human pneumoviruses in their native hosts are more likely to reflect the pathogenesis of natural hRSV infection, and experimental infection of calves with bRSV and of mice with PVM result in clinical disease and extensive pulmonary pathology. These animal models have not only been of value in studies on mechanisms of immunity to and the pathogenesis of pneumovirus infections but have also been used to evaluate hRSV vaccine concepts. Furthermore, the similarities between the epidemiology of bRSV in calves and hRSV in infants and the high level of genetic and antigenic similarity between bRSV and hRSV, make the calf model of bRSV infection a relevant model for preclinical evaluation of hRSV vaccine candidates which contain proteins that are conserved between hRSV and bRSV.

## Highlights

• Respiratory syncytial virus (RSV) is a major cause respiratory disease, worldwide.
• Paediatric and elderly populations are most vulnerable to severe disease.
• Vaccine development has been hampered by the experience of vaccine-enhanced disease.
• Animal models do not necessarily predict vaccine efficacy and safety.
• The strengths and limitations of animal models of RSV infection are summarised.

## 1 Introduction

Human respiratory syncytial virus (hRSV) is the commonest cause of lower respiratory tract infection (LRTI) in children, worldwide, causing disease in an estimated 34 million children, >3 million hospitalizations, and 66,000–199,000 deaths in children under 5 years, each year [1]. Most children are infected during the first year of life, and all have been infected by their second year. Although there is limited viral antigenic variation, the duration of immunity induced by hRSV is short-lived and recurrent infections occur throughout life [2]. The peak incidence of severe disease is in infants 2–7 months of age. However, hRSV also contributes to excess mortality in the elderly [3], [4] and severe hRSV disease occurs in immunosuppressed individuals of any age [5]. The economic impact of hRSV disease in adults is estimated to be greater than that of influenza in relation to numbers of days lost from work [6], [7]. There is no effective anti-viral therapy or licensed hRSV vaccine. However, monoclonal antibody (mAb) prophylaxis is effective in reducing hRSV hospitalisations by 55% in infants at increased risk of severe disease [8].

Hurdles to vaccine development include the need to vaccinate at an early age when maternal antibodies are present, the failure of natural infection to prevent reinfection, a history of vaccine-enhanced disease in young children given a formalin-inactivated (FI) hRSV vaccine [9], and the lack of animal models that fully reproduce the pathogenesis of hRSV infection in man. Since hRSV infection does not prime for enhanced disease, live attenuated vaccine candidates are considered safe, although their protective efficacy may not be very durable. Development of other vaccines for paediatric use is hampered by the difficulty of reliably demonstrating their safety and efficacy in preclinical studies.

## 2 HRSV infection in man

HRSV is mainly transmitted by large particle aerosol or direct contact. Initial viral replication occurs in the nasopharynx with an incubation period of 4–5 days, and can be followed by spread to the LRT. Disease severity ranges from that of a mild common cold to bronchiolitis, with airway obstruction and hypoxia in infants, wheezing, and pneumonia [4]. Apnea is seen in 1–24% of hRSV-infected infants [10], and severe hRSV bronchiolitis is associated with subsequent episodes of wheezing which can persist until ∼11 years of age [11].

**Fig. 1:**
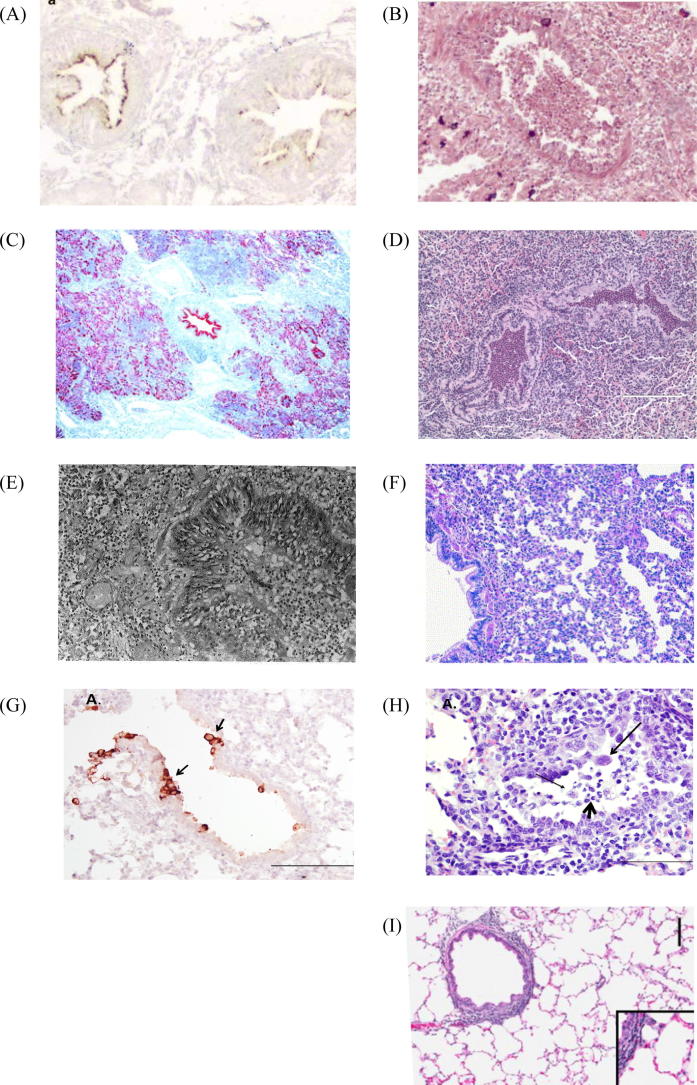
(A) Localisation of human (h)RSV antigen in bronchiolar epithelial cells of a child with an acute hRSV infection. HRSV antigen-positive cells are brown; (B) section of lung from a child with an acute hRSV infection showing acute bronchiolitis in a medium-sized airway with intraluminal cellular debris and inflammatory cells (A and B are adapted from [12], reprinted by permission from Macmillan Publishers Ltd: Modern Pathology, 20:108–119, Johnson et al., copyright 2007); (C) localisation of bovine (b)RSV antigen in bronchial, bronchiolar, and alveolar cells of a calf experimentally infected with bRSV, 6 days previously. BRSV antigen-positive cells are red (adapted from [114], reprinted from The American Journal of Pathology, 161:2195–2207, copyright 2002, with permission from Elsevier); (D) section of lung from a calf, inoculated intranasally and intracheally 6 days previously with bRSV, showing bronchiolitis with intraluminal cellular debris and inflammatory cells, and interstitial pneumonia; (E) section of chimpanzee lung, naturally infected with hRSV, showing alveolar and interstitial pneumonitis and bronchitis (reprinted from [25], J Com Pathol, 110:207–212, copyright 1994, with permission from Elsevier; (F) section of lung from an infant rhesus monkey infected by aerosol with hRSV, 7 days previously, showing mild lymphocytic and histiocytic inflammatory cell infiltrates in the walls and lumens of the terminal conducting airways, and in the septa and lumens of adjacent alveoli (adapted from [45], reprinted from Vaccine, 23:2928–2942, copyright 2005, with permission from Elsevier); (G) section of lung from a lamb infected by aerosol with hRSV, 8 days previously, showing viral antigen within epithelial cells lining the bronchioles (brown cells); (H) section of lung from a lamb infected by aerosol with hRSV, 8 days previously, showing bronchiolitis with degenerate/necrotic individual epithelial cells (thin arrow), occasional syncytial cells (long arrow), accumulation of degenerate neutrophils (short arrow), and occasional macrophages (G and H adapted from [59]; (I) section of lung from a cotton rat infected 5 days previously with hRSV, showing mild peribronchiolitis (adapted from [70], reprinted from Vaccine, 31:306–312, copyright 2013, with permission from Elsevier).

HRSV replicates primarily in ciliated airway epithelial cells and type I and type II alveolar pneumocytes (Fig. 1A) [12]. Histopathological changes of fatal cases of hRSV infection are characterised by peribronchiolar and perivascular mononuclear cell accumulations, interstitial pneumonia, bronchiolar epithelial cell necrosis, and occlusion of the bronchiolar lumen by desquamated epithelial cells, macrophages, neutrophils, fibrin, and mucin, resulting in airway obstruction (Fig. 1B) [12]. Cells in the peribronchial areas of the lung of a child with hRSV who died from an unrelated cause, consisted of monocytes, CD3^+^CD4^−^CD8^−^ T cells and CD3^+^CD8^+^ [12]. However, CD4^+^ and CD8^+^ lymphocytes were rare in lungs of fatal hRSV LRTI [13]. Neutrophils predominate in bronchoalveolar lavage (BAL) from infants with hRSV bronchiolitis [14], [15], and respiratory secretions contain high levels of proinflammatory cytokines (e.g. tumour necrosis factor-α (TNFα), interleukin-6 (IL-6), IL-1α), CXC/CC chemokines (e.g. IL-8, MIP-1α, MCP-1 and RANTES) [16], and interferon (IFN)γ, IL-4, IL-5, IL-10, IL-9 and IL-17 [17], [18]. There is an association between severity of hRSV LRTI and polymorphisms in a number of host response genes (e.g. IL-8 promoter, and CC chemokine receptor 5 (CCR5), which binds Mip-1α and RANTES) [19], [20], suggesting that a robust inflammatory response contributes to hRSV pathogenesis. However, intrinsic properties of different virus strains may also contribute to variations in disease severity [21].

## 3 Animal models of RSV

Animal models of hRSV include those in which hRSV is used to infect non-human mammalian hosts, and those in which non-human pneumoviruses are studied in their natural host. The main non-human mammalian hosts that have been used are non-human primates, cotton rats, mice, and lambs. Apart from chimpanzees, these animals are semi-permissive for hRSV replication and experimental infection with large doses of virus result in little or no clinical signs of disease. In contrast, natural hosts of non-human pneumoviruses, such as bovine (b)RSV and pneumonia virus of mice (PVM), are fully permissive for virus replication and experimental infection results in disease.

### 3.1 Non-human primates

#### 3.1.1 Chimpanzees

HRSV was first isolated from a chimpanzee in a captive colony of 20 animals, 14 of which had a respiratory illness characterised by coughing, sneezing, and a mucopurulent nasal discharge [22]. The virus, which was originally termed chimpanzee coryza agent (CCA), caused a similar upper respiratory tract (URT) disease in 2 out of 3 chimpanzees after intranasal (IN) inoculation with 10^4^ TCID_50_ of tissue culture-passaged material. The animal that did not develop disease was the only one that had pre-existing hRSV-specific serum antibodies. The virus was transmitted not only between chimpanzees but also from chimpanzees to a laboratory worker.

Chimpanzees experimentally infected with ∼10^4^ pfu hRSV shed large quantities of virus (10^5^ and 10^6^ pfu/ml of nasopharyngeal swab sample and tracheal lavage, respectively), for 6–10 days, which is comparable to that seen in children (Table 1) [23], [24]. Although LRTI has not been seen in experimentally infected chimpanzees, fatal cases of hRSV-associated bronchopneumonia, some of which also involved *Streptococcus pneumoniae*, have been reported in captive chimpanzees in zoos and in habituated chimpanzees in a research station in Côte d’Ivoire [25], [26], [27], [28]. HRSV antigen can be detected in the lungs and histopathological changes of extensive purulent bronchopneumonia and interstitial pneumonia with occasional syncytial cells resemble those in human infants (Fig. 1E) [25]. *S. pneumoniae* has been shown to enhance hRSV infection in differentiated human airway epithelial cells, *in vitro*, and in cotton rats [29].

Chimpanzees have been used to evaluate the virulence and protective efficacy of live, attenuated hRSV vaccine candidates [24], [30], [31]. The replication and genetic stability of the mutant viruses in chimpanzees parallels that in sero-negative children, and the mutants induced protection against subsequent wild-type hRSV challenge in chimpanzees [24], [32], [33], [34]. However, chimpanzees vaccinated with recombinant vaccinia viruses (rVV) expressing the hRSV surface glycoproteins F and G developed only low levels of neutralising antibodies, and were poorly protected against hRSV challenge [24], [35]. These findings contrast with the almost complete protection induced in mice, cotton rats and owl monkeys by the same rVV [24], [35].

#### 3.1.2 African green monkeys

African green monkeys (AGMs), which are semi-permissive for hRSV replication (Table 1), have been used in a number of studies to evaluate hRSV vaccine candidates [36], [37], [38], [39], [40], [41]. Clinical signs of disease are uncommon following hRSV infection, and AGMs develop only mild histopathological changes in the lung [38], [42], [43], The proportion of neutrophils in BAL of monkeys infected with the Memphis M37 (M37) strain of hRSV increased only to 9%, [40], whereas neutrophils are the predominant cell in BAL from hRSV-infected children [14].

Vaccine-enhanced pulmonary pathology has been demonstrated following hRSV challenge of AGMs vaccinated with FI-hRSV [42]. Although FI-hRSV induced partial protection against hRSV replication, animals developed severe peribronchiolar and parenchymal inflammation. Enhanced histopathological changes were also induced in hRSV-infected AGMs that had been vaccinated with FI-herpes simplex virus (FI-HSV), although they were less severe than those in FI-hRSV animals. These findings suggest that an immune response to cell culture components present in the vaccine and the hRSV challenge may have contributed to the enhanced pathology seen in FI-hRSV vaccinated animals.

A comparison of the protective efficacy of a number of different non-replicating hRSV vaccines, all of which had been shown to induce complete protection against hRSV in mice and/or cotton rats, demonstrated varying efficacy in AGMs [40]. The vaccines included purified hRSV F and G proteins in different adjuvants, replication incompetent recombinant vesicular stomatitis virus expressing F or G, replication incompetent recombinant adenovirus (rAdV) expressing F or G, and DNA plasmids encoding F or G, administered on 3 occasions in various combinations. Only IN priming with rAdV-F/G followed by F/G protein boosting induced complete protection in both the URT and LRT. However, F/G proteins formulated in Alhydrogel and CpG induced a significant reduction in virus replication. There was no evidence of enhanced pulmonary inflammation in any of the vaccinated monkeys. These observations highlight the failure of small animal models to predict hRSV vaccine efficacy in AGMs.

#### 3.1.3 Macaques

Three species of macaques (rhesus, cynomolgus and bonnet monkeys) have been experimentally infected with hRSV (Table 1). Although infection does not usually produce clinical signs of disease, mild interstitial pneumonia has been seen following IT or aerosol inoculation of high titre virus (>10^6^ pfu) (Fig. 1F) [44], [45], [46], [47], [48], [49], [50], [51]. Macaques are not very permissive for hRSV replication, and susceptibility to infection does not appear to be influenced by age [52]. Viral loads are usually determined by RT-PCR. However, virus has been isolated from the respiratory tract in some studies, and viral mRNA was detected in alveolar and perivascular sites, but not in ciliated epithelial cells [46], [47], [48].

Vaccine-enhanced pulmonary pathology has been described in macaques [48], [49]. Pulmonary pathology in hRSV infected, FI-hRSV-vaccinated bonnet monkeys was associated with enhanced lung virus load, and characterised by peribronchiolar and perivascular accumulations of macrophages, lymphocytes, neutrophils and eosinophils, alveolitis and interstitial pneumonia [48]. However, the extent of pulmonary pathology in FI-hRSV vaccinated animals was not significantly different from that in monkeys vaccinated with FI-Vero cells, suggesting that sensitisation to non-viral antigens contributed to the vaccine-enhanced pathology. Antibody-mediated enhanced uptake and replication of hRSV in macrophages was proposed as the mechanism for increased lung viral load [53]. However, enhanced pulmonary pathology was not associated with increased virus replication in the lungs of FI-hRSV-immunised AGMs [42] or cynomolgus monkeys [49]. Vaccine-enhanced pulmonary pathology in FI-hRSV vaccinated cynomolgus monkeys was associated with pulmonary eosinophilia, and priming of T cells for production of IL-13, IL-5 and IL-2 [49]. Two vaccinated monkeys developed pulmonary hyperinflation and were moribund, 12 days after hRSV challenge. However, the lungs of these animals did not show overt inflammatory lesions or pulmonary eosinophilia. Following challenge with hRSV, lymphocytes from FI-hRSV-immunised monkeys produced IL-13 in response to stimulation with Vero cell lysate suggesting that sensitisation to non-viral antigens may have contributed to the vaccine-enhanced pulmonary pathology.

#### 3.1.4 Other non-human primates

A number of other non-human primates (NHP) have been used as models of hRSV infection, although all are only semi-permissive for virus replication (Table 1).

HRSV-infected owl monkeys develop serous rhinorrhea, which is less severe than that seen in chimpanzees [32], [54] and virus replicates efficiently in the nasopharynx for 8–17 days [54]. However, IT inoculation with higher doses of hRSV (10^6^ pfu) induced only minor histological lung changes [55]. Owl monkeys resembled chimpanzees in their response to infection with live, attenuated hRSV vaccine candidates [32], and therapeutic administration of human IgG containing virus neutralising (VN) antibodies significantly reduced lung viral load, but did not affect pulmonary pathology [56].

HRSV replicates in the URT of cebus monkeys following IN inoculation, but they do not develop clinical signs of disease [23]. However, following IT inoculation with 10^8^ pfu, monkeys developed mild rhinorrhea and conjunctivitis, one developed coughing, dyspnea, anorexia and lethargy, there was a high virus lung load, and nearly all had evidence of pneumonic lesions by radiography [57]. The lungs of all animals had areas of gross pathological changes, histopathological changes were characterised by interstitial pneumonia, and an abundance of viral antigen was detected in ciliated epithelial cells of the nasal turbinate and bronchioles, and in alveolar cells. However, cebus monkeys do not appear to have been exploited for vaccine evaluation.

Infant baboons inoculated IT with a large dose of virus (10^7.9^ pfu) developed clinical signs of LRTI, with tachypnoea, dyspnoea, and a decrease in blood oxygen saturation (<97%) in a proportion of animals [58]. However, virus titres in BAL were maximal 24 h after inoculation, and declined thereafter. Lungs showed vascular congestion and oedema, and microscopic lesions were characterised by interstitial pneumonia, sloughing of the bronchiolar epithelium, and obstruction of the bronchiolar lumen with inflammatory cells and sloughed epithelial cells. Viral antigen was mainly detected in lung parenchyma, and a few scattered epithelial cells. There was a rapid increase in BAL neutrophils 24 h after infection, which declined by day 3, and was followed by an increase in macrophage numbers in BAL. Further studies are needed to determine the extent to which the pulmonary inflammatory response is the result of virus replication or is a non-specific inflammatory response to the high dose inoculum.

### 3.2 Sheep

Neonatal lambs can be infected with high doses of hRSV (Table 2). Infection with hRSV A2 caused a slight fever and cough, macroscopic lung lesions characterised by multifocal areas of consolidation, and microscopic lesions similar to those of hRSV-infected children [59]. Viral antigen was detected in ciliated bronchial epithelial cells and syncytial cells (Fig. 1G and H). Viral mRNA in BAL increased from low levels at day 3 pi to peak at day 6 pi and was cleared by day 14. Lambs infected with high doses of hRSV M37 developed increased respiratory effort and wheezing [60], [61]. Virus titres in BAL increased 100-fold from day 1 to day 6, and infection resulted in increased numbers of macrophages, CD4^+^ and CD8^+^ cells, and levels of IFNγ, IL-8, MCP-1, MIP-1α, RANTES, and surfactant protein A (SP-A) mRNA increased in the lungs [60], [62].

FI-hRSV vaccinated lambs were protected against virus infection and the development of macroscopic lesions [63]. Although there was a more extensive peribronchiolar accumulation of cells in vaccinated lambs, other features of pulmonary pathology were significantly reduced, and there was no evidence of exacerbation of disease. The effect of maternal immunisation on the levels of hRSV-specific antibodies and induction of resistance to hRSV infection in their offspring has been evaluated [64]. Maternal antibodies are transferred to lambs via colostrum and immunisation of pregnant ewes with hRSV F protein in adjuvant induced high levels of virus neutralising (VN) antibodies in serum and colostrum. Lambs receiving colostrum from vaccinated ewes had VN titres (VNT) about 50-fold higher than those in lambs born to non-vaccinated ewes [64]. Following hRSV challenge of lambs at 2–3 days of age, lung viral loads were reduced by ∼70% and there was a significant reduction in pulmonary pathology in lambs born to vaccinated ewes compared with controls. These studies suggest that maternal immunisation is a safe and effective approach to increase protection against hRSV LRTI in young infants.

### 3.3 Rodents

Cotton rats and mice have been used extensively as models of hRSV infection and have provided insights into mechanisms of immunity to and the pathogenesis of hRSV infections [65], [66].

#### 3.3.1 Cotton rats

Although cotton rats are semi-permissive for virus replication, they are about 100-fold more permissive than BALB/c mice per inoculum dose of virus, but they do not develop clinical signs of disease (Table 2) [67]. Following IN inoculation, hRSV replicates to high titres in the nose and lungs, but to lower titres in the trachea. Cotton rats are susceptible to infection throughout life, but virus replication is greater and persists for longer in the nasal passages of 3 day-old rats than in older animals. Viral antigen can be detected in ciliated epithelial cells of the nose and lungs, but not trachea [68], [69], [70], and induces an increase in IFNγ, IL-10, IL-6, MCP-1 and growth-regulated oncogene (GRO) (IL-8 homologue) mRNA in the lungs [71], and histopathological changes characterised by a desquamative, exudative rhinitis, and a mild proliferative bronchiolitis (Fig. 1I) [68], [72]. Virus loads are greater and virus persists for longer in cotton rats immunosuppressed by cyclophosphamide, and animals develop a more severe pulmonary inflammatory response characterised by foamy macrophages, and bronchial and tracheal intraluminal debris [73], [74].

There is a direct correlation between levels of VN serum antibodies associated with protection in cotton rats and infants. Serum VNT of >1:380 are protective in passively immunised cotton rats and similar levels of maternally derived serum antibodies (MDA) in human infants <2 months old correlate with increased resistance to hRSV LRTI [75]. The cotton rat has played an important role in the development of mAb prophylaxis for the prevention of LRTI in infants at high risk of severe hRSV disease [75], [76], and is a well-established model of FI-hRSV vaccine-enhanced disease. Although FI-hRSV vaccination reduced hRSV replication, cotton rats developed an enhanced peribronchiolitis, alveolitis and interstitial pneumonitis [77]. Whereas peribronchiolitis is thought to be a normal component of the immune-mediated resolution of hRSV infection, alveolitis is considered to be the primary marker of vaccine-enhanced disease in the cotton rat model [65]. However, the severity of alveolitis in FI-hRSV-vaccinated cotton rats is associated with priming of T cells specific for non-viral antigens, and this response is potentiated by hRSV antigens [72]. This is reminiscent of the response of T cells from FI-hRSV vaccinated macaques, but not of those from monkeys vaccinated with FI-measles virus, which responded to vero cell antigen following hRSV challenge [49]. These findings indicate that caution should be used when extrapolating results from this model to assess the safety of human vaccine candidates.

#### 3.3.2 Mice

Although inbred mouse strains vary in their susceptibility to experimental hRSV infection [78], the BALB/c mouse, which shows intermediate susceptibility, has been widely used to study the pathogenesis of and mechanisms of immunity to hRSV [66]. Following IN inoculation, hRSV replicates in the nasal passages and lungs (Table 2) [78], [79]. Although mice are susceptible to hRSV infection throughout life, older mice are more susceptible than younger animals [80], [81]. High (>10^6^ pfu) doses of hRSV are needed to induce clinical signs of disease, which is characterised by weight loss, ruffled fur and a hunched posture. Microscopic lesions consist of perivascular and peribronchiolar accumulations of mononuclear cells [80] and increased mucus production. Levels of pro-inflammatory cytokines and chemokines are increased during the acute phase of infection [82], [83], [84]. Both CD4^+^ and CD8^+^ T cells play a role in virus clearance and contribute to disease. Depletion of either subset, or both, prolongs virus infection and reduces weight loss, illness, and pulmonary pathology [85]. These observations contrast with the prolonged viral shedding, severe respiratory disease, and giant cell pneumonia seen in immunosuppressed humans [4].

Laboratory strains of hRSV replicate primarily in type I alveolar pneumocytes. However, there is extensive replication of hRSV in bronchiolar epithelial cells in mice deficient in type I IFN pathways [82] suggesting that the mechanisms that hRSV employs to evade type I IFN responses are poorly effective in the mouse. Some clinical isolates also infect bronchial epithelial cells in mice, and clinical isolates vary in their virulence in mice [86]**.** HRSV infection of mice induces airway obstruction and airway hyper-responsiveness (AHR) in response to methacholine [83], and the mouse model has been used to determine the immunological mechanisms that result in virally-induced AHR (reviewed [11], [87]).

FI-hRSV vaccine-enhanced disease in mice is associated with priming of a Th2 response and is characterised by pulmonary eosinophilia, increased pulmonary pathology, mucus hypersecretion, weight loss, increased airway obstruction and AHR, and a reduction in lung viral load [88], [89]. Depletion of CD4^+^ cells or IL-4 and IL-10 abrogates vaccine-enhanced pathology [90], [91]. Low avidity, non-neutralising antibodies induced by FI-hRSV vaccination of mice have also been shown to contribute to enhanced AHR and pulmonary pathology [92], [93]. As seen in other animal models, immune responses to non-viral antigens contribute to vaccine-enhanced disease in the mouse [72], [94], [95]. However, a study in which vaccine virus was produced in serum-free medium in different cells from that used for challenge virus, showed that FI-hRSV primed both Th1 and Th2 responses. Th2 responses mediated enhanced pulmonary pathology, pulmonary eosinophilia, mucus hypersecretion and AHR, whereas TNF-α contributed to airway obstruction and weight loss; eosinophils did not contribute to any disease parameters associated with FI-hRSV vaccine-enhanced disease in mice [89].

The protective effects of mAbs, *in vivo*, was first demonstrated in the mouse model of hRSV [96], and led to the development of a protective, humanised mAb specific for the RSV F protein [97] and ultimately to the development of Palivizumab prophylaxis. The mouse model of hRSV is useful for the negative selection of vaccine candidates. However, since there are many differences in innate and adaptive immune responses between mice and humans [98], the responses of mice to vaccination may differ substantially from those which occur in higher species. For example, recombinant vaccinia Ankara (MVA) expressing the hRSV F or G proteins completely protects mice from hRSV infection [99], but did not induce protection in macaques [100].

Infection of a humanised mouse model, which lacks functional CD8^+^ T cells, with 10^6^ pfu of hRSV line 19 induced weight loss and pulmonary pathology characterised by peribronchiolar inflammation, a predominance of neutrophils in BAL, and enhanced mucus production [101]. The predominance of neutrophils in BAL is similar to that in hRSV-infected children, and contrasts with hRSV-infection of BALB/c mice, where the proportion of neutrophils in BAL is usually ∼10% [102]. A humanised mouse model may be a more relevant model for evaluating hRSV vaccine candidates than conventional mice.

#### 3.3.3 Ferrets

Following IN infection, HRSV replicates in the nasal passages of ferrets of all ages, but only in the lungs of infant animals (Table 2) [103], [104]. However, replication of hRSV in the LRT of adult ferrets, with viral antigen seen in ciliated epithelial cells of the trachea and bronchi has been demonstrated following IT inoculation with a low passage, clinical isolate of hRSV [69]. Viral antigen could be detected in bronchiolar epithelial cells only in immunosuppressed ferrets, in which virus replicated to higher titres and clearance was delayed [69]. Although ferrets did not develop clinical signs of disease, sporadic neutrophils were seen in the tracheal epithelium, and the bronchiolar and alveolar lumina. The possible transmission of infection from infant ferrets to their mothers [104], suggests that ferrets could be used to determine the ability of vaccines to block virus transmission.

#### 3.3.4 Other rodents

Although guinea pigs can be experimentally infected with hRSV, and develop histopathological changes characterised by bronchiolar epithelial necrosis, peribronchial mononuclear and neutrophil infiltrates, and necrotic cells and debris in some bronchioles, they have not been used to evaluate hRSV vaccine efficacy (Table 2) [105].

Syrian hamsters are semi-permissive for hRSV and although virus replicates in the URT and LRT, they are ∼100-fold less permissive than cotton rats (Table 2) [106], and infection does not produce clinical signs of disease or microscopic evidence of pulmonary pathology [106]. Hamsters have been used to evaluate the protective efficacy of recombinant parainfluenza viruses expressing hRSV surface glycoproteins [107], [108].

Chinchillas can also be infected with high doses of hRSV, and a small inoculum volume restricts infection to the URT (Table 2) [109]. Virus was isolated from the nasopharynx for at least 8 days, and infection resulted in mild multifocal neutrophilic and lymphocytic infiltrates of the nasal, goblet cell hyperplasia, hypersecretion of mucus in the Eustachian tube lumen, a mild retraction of the tympanic membrane and a decrease in middle ear pressure. Virus was detected in ciliated epithelial cells of the nasopharynx, nasoturbinates, and Eustachian tube [110]. The chinchilla may be a useful model for evaluation of vaccines to control hRSV infection of the URT, and to investigate the mechanisms by which hRSV infection predisposes to middle ear bacterial infections in children.

## 4 Non-human pneumovirus animal models

An advantage of studying non-human pneumoviruses in their native host is that the host is fully permissive. Studies of bovine (b)RSV [111], and pneumonia virus of mice (PVM) [112] in their natural host have increased our understanding of pneumovirus pathogenesis and support vaccine development concepts.

### 4.1 BRSV in calves

BRSV is a leading cause of respiratory disease in young calves, worldwide [113]. The epidemiology and pathogenesis of naturally occurring bRSV infections are similar to those of hRSV in children. The peak incidence of severe disease is in calves <6 months of age and, as seen in children, MDA do not protect against bRSV infection, but the incidence and severity of respiratory disease is inversely related to the level of maternally-derived VN antibodies [114]. The severity of infection can range from subclinical, to severe with LRTI, and death. BRSV-infected calves are also susceptible to secondary bacterial infections, although some outbreaks can be attributed to bRSV alone. In the UK, 70% of calves have been infected by 9 months of age, and although cattle can be reinfected with bRSV, disease following reinfection is usually subclinical or mild. However, primary bRSV infections causing severe disease have been reported in older cattle [115]. The incubation period is estimated to be between 2 and 5 days, and clinical signs include mild depression, nasal seromucoid discharge, cough, tachypnea, dyspnoea, and fever, with abnormal breath on ausculation. Macroscopic lung lesions have a mainly cranioventral distribution and are characterised by areas of consolidation. BRSV replicates primarily in ciliated airway epithelial cells and type II pneumocytes [116] (Fig. 1C), and induces microscopic lesions of bronchointerstitial pneumonia, epithelial necrosis, an exudative or proliferative alveolitis, and occasional syncytial cells (Fig. 1D). The lumen of the bronchi and bronchioles are obstructed by cellular debris consisting of neutrophils, desquamated epithelial cells, macrophages, and mucin [117].

Although bRSV is genetically and antigenically closely related to hRSV (Table 3), bRSV is highly restricted in non-human primates [118]. The similarities in the epidemiology and pathogenesis of hRSV and bRSV infections, and the close antigenic relationship between these viruses, make bRSV infection in calves a relevant animal model of hRSV. Initial attempts to induce clinical signs of respiratory disease in calves experimentally infected with bRSV yielded inconsistent results [111]. However, reproducible clinical signs of disease have been produced in calves following inoculation with ∼10^4^ pfu of low- or non-cell-culture-passaged bRSV (Table 4) [119], [120], [121], [122]. The calf allows daily sampling of the URT, and virus replication in the LRT and the inflammatory response can be analysed in BAL or in the lung tissue at post-mortem examination. Virus titres in the URT peak at ∼10^4^ pfu/ml and at ∼10^5^ pfu/ml in BAL, and virus is normally cleared by day 8–9. Clinical signs of disease usually appear between day 5 and 6 and are maximal at day 7–8. Macroscopic and microscopic lesions of experimentally infected calves are similar to those observed in naturally occurring bRSV infections (Fig. 1), and can be induced in the absence of other respiratory pathogens. Experimentally infected calves have a significant decrease in arterial oxygen tension and a diffuse pattern of patchy consolidation can be detected by thoracic radiography [120], [121]. BRSV infection induces increased levels of pro-inflammatory cytokines and chemokines, and a marked increase in neutrophils in BAL similar to that in children with hRSV bronchiolitis [117], [123], [124], [125].

CD8^+^ T cells are important in virus clearance of bRSV from both the URT and LRT and, in contrast to T-cell depleted hRSV-infected mice, prolonged bRSV infection in CD8^+^-depleted calves is associated with more severe pulmonary pathology [126]. Neutralising mAbs to antigenic sites corresponding to amino acids 255–275 (antigenic site II) and 417–438 (antigenic site IV) of the F protein neutralise both hRSV and bRSV, and passive immunisation with bovine mAbs specific for these antigenic sites protect calves from bRSV LRTI [127], [128].

There is evidence of vaccine-enhanced disease in calves following a natural bRSV infection. Severe disease with a high mortality was seen in 34% of 7 month-old calves vaccinated with a commercial β-propiolactone-inactivated bRSV vaccine, whereas disease in non-vaccinated, 2–3 month-old calves was less severe and no animals died [129]. BRSV antigen was detected in the lungs of the vaccinated calves and lung lesions were characterised by interstitial pneumonia, with bronchitis and bronchiolitis obliterans, pneumocyte type II hyperplasia, syncytial cell, and an infiltration of neutrophils and eosinophils. These features of altered age distribution for severe disease and the pattern of lung pathology were similar to those seen in FI-hRSV- vaccinated infants [9]. Exacerbated disease has also been seen in calves vaccinated IM with a live, attenuated bRSV vaccine administered during a concurrent bRSV infection [130]. However, attempts to experimentally reproduce vaccine-enhanced disease with FI-bRSV have yielded conflicting results. Some studies have demonstrated enhanced disease with pulmonary eosinophilia in vaccinated calves [131], whereas others have not reported an increase in eosinophils [132]. FI-bRSV vaccination has induced varying levels of protection against infection, and either enhanced pulmonary pathology, or an earlier onset of disease followed by a faster resolution and less severe pathology [120], [131], [132], [133]. These differences may be related to the vaccine antigen dose, the level of MDA at vaccination, and/or the interval between vaccination and challenge. An advantage of using the calf as a model of vaccine-enhanced disease is that virus for vaccine production and challenge are grown in bovine cells maintained in calf serum, so there is less likelihood that calves will be sensitised to foreign cell culture antigens.

The calf model of bRSV infection has been used to evaluate the virulence, immunogenicity and protective efficacy of a variety of recombinant bRSV in which various genes have been deleted [134], [135], [136]. The findings from these studies are similar to those seen in chimpanzees infected with analogous mutants of hRSV [31]. Calves have been used to evaluate hRSV vaccine concepts such as DNA vaccines [137], subunit vaccines [138], immunostimulating complexes (ISCOMs) [139], live attenuated viruses [140], recombinant virus vectors [141], [142], and novel adjuvants with inactivated bRSV [143], and to determine the effects of maternal antibodies on vaccination [144]. Calves can also be part of the preclinical assessment of hRSV vaccine candidates which contain proteins that are conserved between hRSV and bRSV. For example, calves vaccinated IN with rAdV expressing a fusion protein consisting of the hRSV F, N and M2-1 proteins and boosted IM with MVA expressing the same antigens were completely protected against subsequent bRSV challenge [145]. These same vaccine vectors can effectively boost immune responses in healthy adult volunteers [146]. Furthermore, the ability to repeatedly sample different regions of the respiratory tract of the same animal during the course of bRSV vaccination and challenge studies could contribute to a greater understanding of mucosal immunity and identification of correlates of protection against bRSV infection.

### 4.2 PVM in mice

PVM was originally isolated form mouse lungs and is a common pathogen of laboratory mouse colonies [147]. Experimental infection of mice with PVM results in disease that varies in severity depending on the virus dose, mouse strain, and age [148], [149], [150]. BALB/c mice are susceptible and C57BL/6 mice are relatively resistant. Following IN inoculation of BALB/c mice with low doses (60–600 pfu) of virulent strains of PVM, virus replicates to high titres in the lung, causing severe morbidity with weight loss, ruffled fur, hunched posture, laboured breathing, and cyanosis, and 100% mortality (Table 4) [151], [152]. Virus replication occurs initially in alveolar cells and subsequently in bronchial epithelial cells [152], [153], and induces an early increase in eosinophils in BAL followed by a predominantly neutrophil response [154]. Lung histopathological changes consist of alveolar epithelial cell apoptosis, bronchial epithelial necrosis, multifocal acute alveolitis, intra-alveolar oedema, haemorrhage, and granulocytic infiltration. The pro-inflammatory chemokines and cytokine responses in the lungs of PVM-infected mice are similar to those of hRSV-infected mice [155]. However MIP-1α, which controls the inflammatory response induced by PVM [156], does not control the inflammatory response induced by hRSV in mice [157]. These findings highlight differences in the pathogenesis of pneumoviruses in a natural host and in an unnatural, semi-permissive animal model. Both CD4^+^ and CD8^+^ T cells contribute to virus clearance, clinical disease and pulmonary pathology in mice infected with sub-lethal doses of PVM. However, CD8^+^ T cells are largely responsible for the cytokine storm [158].

Enhanced disease in FI-PVM-vaccinated mice is associated with a Th2-biased response, production of IL-4, IL-5, IL-13, and pulmonary eosinophilia [159]. In contrast, neutrophils predominate in BAL from mice vaccinated with FI-uninfected cell antigen, and Th2 cytokine levels are low. However, mice vaccinated with FI-PVM had a reduced lung virus load and were partially protected against weight loss, despite the absence of VN antibodies. Sensitisation to non-virus antigens are unlikely to have contributed to enhanced pathology in FI-PVM-vaccinated mice as the challenge virus had been passaged *in vivo*, and was therefore devoid of foreign non-viral antigens that could cross-react with those in the PVM vaccine. Whilst hRSV vaccine approaches can be studied in the mouse model of PVM, and vaccine candidates can be negatively selected, their ability to predict efficacy in higher species is not known. IN vaccination of mice with rAdV expressing either the M or N protein of PVM protected mice against a lethal challenge dose of PVM, although mice developed a transient weight loss [160].

Whilst the advantage of the mouse model of PVM is the ability to study a pneumovirus infection in a natural host, the many differences in the innate and adaptive immune response between mice and humans, the differences in lung anatomy, and the antigenic differences in the viral proteins, limits its relevance to hRSV in humans. Nevertheless, an understanding of the pathogenesis and mechanisms of immunity to PVM can inform studies in higher animal species and man.

## 5 Concluding remarks

The natural progression of hRSV infection in humans, which is probably initiated by exposure to small doses of virus in the URT, is bypassed in the majority of animal models of hRSV infection, where large doses of virus are introduced directly into the lungs. As a result, the innate immune defences in the LRT are rapidly mobilised to deal with a large load of foreign antigen, which will impact on the timing and magnitude of the adaptive immune response. This has consequences for understanding the pathogenesis of hRSV infections and for evaluating the protective efficacy and safety of vaccines. Immunopathology is likely to result from a very high viral load encountering large numbers of virus-specific effector cells and/or poorly neutralising antibodies in the lungs. In the normal course of events, the initial period of virus replication in the URT provides an opportunity for the adaptive immune response to be activated prior to the accumulation of large virus loads in the lungs. Even in non-human pneumovirus animal models, virus is delivered directly to the lungs in order to ensure reproducible LRTI.

Interpretation of findings from animal models of vaccine-enhanced disease has been confounded by sensitisation to non-viral antigens in the vaccine and challenge virus. In order to minimise priming of lymphocytes to non-viral antigens in vaccine formulations that might confound results obtained from challenge of vaccinated animals, the cells and medium components used to produce vaccine preparations and virus challenge should be considered carefully.

The chimpanzee is the only animal host that is fully permissive to hRSV replication, allowing animal-to-animal transmission, and in which infection reliably produces URT disease. However, the close genetic and antigenic relatedness of hRSV and bRSV, and the similarities between hRSV in children and bRSV infection in calves suggest that the calf model has many advantages. However, virulent bRSV is difficult to grow to high titres in cell culture, calves require specialised housing, and the availability of reagents to dissect the immune response is not as extensive as that for mice and humans. Mice and cotton rats can be used for the negative selection of vaccine candidates. However, the extent to which they predict vaccine efficacy in man is not clear; therefore higher animal species should also be used for preclinical evaluation of new vaccine candidates.

## Conflict of interest

The author has no conflict of interest to declare.

## Figures and Tables

**Table 1:** Non-human primate models of hRSV infection.

Species	Age	Virus strain^a^	Inoculum	Route^b^	Viral replication	Clinical signs of disease^c^	Pathology	Reference
Chimpanzees (*Pan troglodytes*)	20–24 mths	hRSV	10^4^ TCID_50_	IN	Virus isolated	URT illness	Not done	[22]
	15–18 mths	hRSV A2	10^3.5^ pfu	IN	High	URT illness	Not done	[23]

Owl monkeys (*Aotus trivirgatus*)	Adult	hRSV A2	10^3.7^–10^6^ pfu	IN or IT	Moderate	Serous rhinorrea	Minor histological changes	[32], [54], [55]

Baboons (*Papio cynocephalus anubis*)	4 wks	hRSV A2	10^7.9^ pfu	IT	Virus tires declined from day 1	Tachypnoea & dyspnoea	Gross changes of vascular congestion & oedema; Interstitial pneumonia, sloughing of bronchiolar epithelium, obstruction of the bronchiolar lumen	[58]

Cebus monkeys (*Cebus apella*, *Cebus albifrons*)	6–20 mths	hRSV A2	10^8^ pfu	IT	Moderate	Rhinorrhea & conjunctivitis	Extensive interstitial pneumonia, alveolitis, syncytial cells	[57]

African green monkeys (*Cercopithecus aethiops*)	Adolescent & adult	hRSV A2	10^3^ pfu	IN & IT	Moderate	Rhinorrhea, sneezing, & wheezing	Patchy inflammation in terminal bronchioles, interstitium & alveoli, syncytial cells; Slight increase in neutrophils in BAL	[42]
	Not reported	hRSV M37	10^5.8^ pfu	IN	Moderate	None	Not done	[40]

Rhesus macaques (*Macaca mulatta*)	1 wk	hRSV A2	10^3.2^ pfu	IN	Low	None	Not done	[23]
	Young adults	Rhesus-adapted hRSV Long	10^5.7^ TCID_50_	IN	Moderate	Not reported	Not done	[50]
	1.5–5.5 mths	Clinical isolate hRSV	10^5.7^–10^7^ TCID_50_	Aerosol	Low	Slight fever & increased RR	Foci of broncho-interstitial pneumonia	[44], [45]
	Young adults	Macaque-adapted clinical isolate	10^5^ pfu	IN	Moderate	None	Not done	[51]

Bonnet monkeys (*Macacca radiata*)	Juveniles	hRSV Long	10^6^–10^6.7^ pfu	IT or intra-bronchial	Moderate	Tachypnoea & chest retractions	Foci of broncho-interstitial pneumonia & alveolitis	[46], [47], [48]

Cynomolgus monkeys (*Macacca fascicularis*)	8–15 mths	Macaque-adapted hRSV	10^6^ TCID_50_	IT	Low	None	Foci of broncho-interstitial pneumonia, alveolitis, syncytial cells	[49]
	4 mths, 1 yr, adults	hRSV	10^5^ TCID_50_	IN	Low	None	Not done	[52]

a Animals were infected with different isolates of human respiratory syncytial virus (hRSV).

b Animals were inoculated by the intranasal (IN), intra-tracheal (IT) or a combination of both IN and IT routes.

c URT = upper respiratory tract.

**Table 2:** Lamb and rodent models of hRSV infection.

Animal	Age	Virus strain^a^	Inoculum	Route^b^	Viral replication^c^	Clinical signs of disease	Pathology	Reference
Neonatal lambs	2–3 days	hRSV A2	10^8^ pfu	Intra-bronchial or aerosol	RSV mRNA detected by RT-PCR	Increased temperature, coughing	Multifocal areas of pulmonary consolidation. Mild to moderate bronchiolitis, interstitial pneumonia	[59], [62]
	2–3 days	hRSV M37	10^8^ pfu	Aerosol	Moderate in LRT	Increased respiratory effort wheezing	Multifocal areas of pulmonary consolidation. Bronchitis/bronchiolitis, interstitial pneumonia, alveolitis	[61]

BALB/c mice	4–32 wks	hRSV A2	10^4.2^–10^7^ pfu	IN	Moderate	Weight loss at high doses	Mild to moderate bronchiolitis	[80], [81]

Cotton rats	Neonatal to adults	hRSV Long	10^4^ pfu	IN	Moderate	None	Mild, proliferative bronchiolitis	[68]

Ferrets	Neonatal to adults	hRSV Long	10^3.5^ pfu	IN	High in nose	None	Mild, focal desquamative rhinitis	[104]
	9–12 mths	hRSV-A clinical isolate	10^5^ TCID_50_	IT	Moderate in LRT	None	Sporadic neutrophils in tracheal epithelium, bronchiolar & alveolar lumina	[69]

Guinea pigs	Adult	hRSV Long	10^3.6^ pfu	IN	Moderate	None	Bronchiolar epithelial necrosis, peribronchial mononuclear & neutrophil infiltrates	[105]

Chinchillas	Juvenile	hRSV A2	10^7^ pfu	IN	Moderate	Ruffled fur & lethargy	Mild inflammation of nasopharynx and eustachian tube	[109], [110]

Syrian hamsters	3 wks	hRSV A2	10^6^ pfu	IN	Moderate	None	None	[106]

a Animals were infected with different isolates of human respiratory syncytial virus (hRSV).

b Animals were inoculated by the intranasal (IN) route.

c LRT = lower respiratory tract.

**Table 3:** Amino acid sequence identity between the protein of hRSV subgroup A, hRSV subgroup B (hRSV-B), bovine (b)RSV and pneumonia virus of mice (PVM).

Viral protein		% Amino acid sequence identity		
		hRSV-B	bRSV	PVM
NS1	Non-structural protein 1	87	69	16
NS2	Non-structural protein 2	92	84	20
N	Nucleoprotein	96	93	60
P	Phosphoprotein	91	81	33
M	Matrix protein	91	89	42
SH	Small hydrophobic protein	76	38	23
G	Attachment glycoprotein	53	30	12
F	Fusion protein	89	81	43
M2-1	Matrix protein 2–1	92	80	43
M2-2	Matrix protein 2–2	72	42	10
L	RNA polymerase	93	84	53

**Table 4:** Models utilising non-human pneumoviruses.

Animal	Age	Virus strain^a^	Inoculum	Route^b^	Viral replication^c^	Clinical signs of disease	Pathology	Reference
Calves	3–6 wks	bRSV Snook	10^3.7^–10^4^ pfu	IN & IT or aerosol	Moderate	Nasal discharge, raised respiratory rate, cough, fever, dyspnoea	Macroscopic lung lesions. Broncho-interstitial pneumonia, epithelial necrosis, exudative or proliferative alveolitis, occlusion of bronchiolar lumina	[122], [135]

BALB/c mice	Adult	PVM J3666	120 pfu	IN	High in LRT	Weight loss, hunched posture, ruffled fur, laboured breathing, cyanosis, death	Multifocal acute alveolitis, intra-alveolar oedema, scattered haemorrhage, moderate granulocytic infiltrates	[152]
	Adult	PVM 15	300–600 pfu	IN	High in LRT	Weight loss, hunched posture, ruffled fur	Broadly dispersed lesions with cellular infiltrates in the alveolar space and surrounding tissues & oedema	[151], [153]

a Animals were infected with bovine respiratory syncytial virus (bRSV) or pneumonia virus of mice (PVM).

b Animals were inoculated by the intranasal (IN), or a combination of both IN and intratracheal (IT) routes.

c LRT = lower respiratory tract.
